# Minimising harms of tight glycaemic control in older patients with type 2 diabetes

**DOI:** 10.4102/phcfm.v16i1.4857

**Published:** 2024-12-18

**Authors:** Wade Thompson

**Affiliations:** 1 Department of Anaesthesiology, Pharmacology and Therapeutics, Faculty of Medicine, University of British Columbia, Vancouver, Canada

**Keywords:** type 2 diabetes, older adults, glycaemic targets, hypoglycaemia, therapeutics

## Abstract

In older adults with type 2 diabetes (T2DM), tight glycaemic control (HbA1c < 7%) can result in more harm than benefit, especially when using insulin or sulfonylureas. Older adults are at higher risk for adverse drug events, especially hypoglycaemia, which may cause falls, confusion and hospitalisations. This Therapeutic Letter evaluates the risks of tight glycaemic control in older adults with T2DM, focusing on deprescribing diabetes medications in those over 65, especially those with multimorbidity and polypharmacy. It assesses the evidence from clinical trials and guidelines, with a focus on preventing hypoglycaemia and improving patient-centred care through relaxed HbA1c targets. Large randomised controlled trials show that intensive glycaemic control (HbA1c ≤ 7%) does not reduce cardiovascular risk, but increases hypoglycaemia and mortality, particularly in older adults. Instead, glycaemic targets should be adjusted based on the patient’s overall health and life expectancy. Deprescribing may be considered, starting with drugs most likely to cause hypoglycaemia (sulfonylureas or insulin). Regular reassessment and patient involvement in creating individualised treatment plans are essential.

## Vignette

*Your 81-year-old patient has a new HbA1c result of 6.6%. He has had type 2 diabetes for 15 years, and he also has chronic obstructive pulmonary disease, osteoarthritis, hypertension, and had a myocardial infarction 10 years ago. He takes 12 daily medications, including metformin, sitagliptin, and glyburide. Having ordered the HbA1c test, how do you respond to the result?*

## Introduction

The prevalence of diabetes is increasing in Africa, and by 2021, 5.2% of adults (20–79 years) had diabetes. In older adults, this increases to approximately 9% in women and 8% in men.^1^ South Africa and Nigeria have the largest number of people living with diabetes, while Tanzania and Zambia have the highest prevalence.^1^ However, older adults with type 2 diabetes (T2DM) often have several other chronic health problems, functional limitations, frailty and polypharmacy. This increases the risk of adverse drug events,^2^ especially hypoglycaemia – an important cause of falls, confusion, emergency department visits and hospitalisation.^3,4,5^ In Africa, metformin, a sulphonylurea and insulin are the most commonly prescribed medications. Newer drugs such as repaglinide are usually only available in the private sector.

## Lower glycaemic targets unnecessary and harmful for older adults

There is no evidence to specify ‘ideal’ HbA1c targets in older adults (≥ 65 years). Aiming for a specific number or threshold is complicated by the continuous variability of HbA1c in a population (similar to haemoglobin, creatinine or body weight). Individuals also show day-to-day biological variability and measurement error in HbA1c.^6^

Large randomised controlled trials (RCTs) examined the effects of treating to intensive glycaemic targets (e.g. HbA1c ≤ 7% or ≤ 6.5%), versus treating to relaxed targets (~7.5% – 8.5%) among people with longstanding T2DM.^7,8,9^ These RCTs were conducted in groups whose mean age ranged from 60 to 66 years. They mainly tested the use of metformin in combination with sulfonylureas or insulin to achieve targets. Aiming for intensive glycaemic targets did not reduce the risk of cardiovascular (CV) events versus more relaxed HbA1c targets.^7,8,9^ But compared with a relaxed target, achieving HbA1c ≤ 7% increased the risk of severe hypoglycaemia^7,8,9^ and it increased the risk of premature mortality in the Action to Control Cardiovascular Risk in Diabetes (ACCORD) trial.^8^

Targeting an HbA1c ≤ 7%, especially with sulfonylureas or insulin, is now widely considered ‘overtreatment’ for older adults.^10,11,12^ If choosing a target-based approach, current organisational guidelines consistently recommend relaxed glycaemic targets for most older adults with T2DM, and avoidance of sulfonylureas or insulin.^10,11,12^ For example, the Society for Endocrinology, Metabolism and Diabetes of South Africa recommends individualised HbA1c targets. They suggest a target between 7.1% and 8.5% for those who are ‘frail, have a high level of dependency, multiple comorbidities, severe cardiac or vascular disease, advanced renal disease, limited life expectancy or hypoglycaemic unawareness’.^12^

## Preventing hypoglycaemia should be a central clinical goal of type 2 diabetes management in older adults

Recent evidence raises further questions about the appropriateness of treating to intensive HbA1c targets.^10,11,12^ Large RCTs of sodium-glucose cotransporter-2 inhibitors (SGLT2i) and glucagon-like peptide-1 receptor agonists (GLP1RA) for T2DM showed that CV events were reduced with achieved HbA1c values in the mid 7% range, among patients with a mean age in the early 60s.^13,14^ This suggests that CV benefits may be independent of HbA1c lowering. Offering SGLT2i or GLP1RA to older people with T2DM reflects the clinical goal of reducing CV morbidity, instead of a surrogate number. New medications such as SGLT2i and GLP1RA are usually only available in the private sector and affordable for those with medical insurance. Costs of SGLT2i can sometimes be reduced by dose-splitting.^15^

However, very limited evidence is available about the effectiveness and safety of SGLT2i or GLP1RA in people ≥ 75 years, or who have multimorbidity or frailty. In RCTs of SGLT2i or GLP1RA that included patients with T2DM, about 10% of participants were ≥ 75 years.^13,14,16,17,18,19^ Thus, even sub-analyses of landmark trials that claim safety in older adults included few people older than 75.^19,20,21,22^ In frail older people, relevant adverse effects of SGLT2i include dehydration, urinary tract infection, euglycaemic diabetic ketoacidosis and orthostatic hypotension; and from GLP1RA, weight loss or gastrointestinal adverse effects.^11^

## Reassessing diabetes drugs

What if a patient has used the same diabetes medication(s) for years? As chronic conditions accrue, and susceptibility to adverse effects increases with age, overall health status may decline, requiring reassessment.^23^ To assess whether medication changes are needed, consider:

- Is treatment to a target HbA1c congruent with outcomes important to this individual?
- How different is the actual HbA1c from what is medically appropriate for this individual?
- Are adverse effects from diabetes drugs present, or is there increased risk for hypoglycaemia (previous hypoglycaemia, hypoglycaemia unawareness, malnutrition, cognitive impairment, impaired renal function)?
- Is the individual taking a sulfonylurea or insulin?
- Are any medications particularly burdensome or expensive?

## Evidence and guidance for deprescribing

When a patient’s diabetes medications do not align with health goals or the harms outweigh benefits, consider whether deprescribing is appropriate. Three systematic reviews examined evidence on deprescribing of diabetes medications, primarily in people at risk for hypoglycaemia.^24,25,26^ Deprescribing does not appear to increase adverse events or risk clinically significant changes in glucose control. A clinical practice guideline and two-page decision support tool from deprescribing.org can help guide decisions and plans.^27^

## Practical considerations for deprescribing

Start with drugs most likely to cause hypoglycaemia: sulfonylureas or insulin.^27^ Develop individualised deprescribing plans together with patients. Most people value being involved in planning.^28^ Decide whether to:

- stop a drug
- gradually taper it to the minimum available dose before stopping
- reduce the dose, or
- switch to a drug with a more favourable benefit versus harm profile.

There is no evidence from RCTs to identify one best approach.^27^ The most appropriate choice for any individual may depend on actual drug dose(s), patient context (e.g. baseline glycaemic control, risk of harm), expected longevity, and patient goals and preferences. Even when clinicians consider deprescribing as desirable, some patients may view a therapeutic decision to relax glycaemic targets as ‘giving up’.^29^ Explaining clearly the reasons to de-intensify diabetes treatment can help to engage patients and caregivers in a decision to deprescribe.^30^

Possible discussion points, depending on the patient’s health context, include^27,31^:

- ‘Tight’ glycaemic control risks dangerous hypoglycaemia, without compensatory benefits.
- Ageing makes people more susceptible to adverse drug effects (e.g. confusion, falls and fractures caused by hypoglycaemia). What worked well before may no longer be optimal.
- Periodic re-evaluation of diabetes care is always a good clinical practice.

Patients value a clear monitoring and follow-up plan and clinical support throughout the deprescribing process.^28^ They want to know what to expect and what to do if they experience problems. This includes understanding that they can restart a diabetes medication or switch to another drug, when clinically appropriate (e.g. if symptomatic hyperglycaemia occurs).^28^

Blood glucose results can be monitored during follow-up, along with symptoms of hypo- or hyperglycaemia. The deprescribing.org guideline for reducing diabetes medications suggests follow-up 1–2 weeks after changes in therapy to review capillary or venous blood glucose, and to assess for symptoms.^27^ Because it equilibrates slowly with blood glucose, re-testing HbA1c is inappropriate until at least 3 months after changes to drug therapy.

**Vignette resolution:** *Your 81-year-old patient has an HbA1c of 6.6%. Reducing potentially dangerous or unnecessary diabetes drugs makes sense. Glyburide increases the risk of hypoglycaemia, while sitagliptin has not been shown to prevent any complication of diabetes. After discussion, you decide together to relax his HbA1c goal, stop glyburide first, re-check his HbA1c after 3 months and then reconsider sitagliptin.*

## Summary and conclusions

- An HbA1c below 7% is likely associated with more harm than benefit in older adults (≥ 65 years) with T2DM taking sulfonylureas or insulin.
- Deprescribing is appropriate when HbA1c is < 7% for older adults with T2DM taking sulfonylureas or insulin. Stop, gradually taper or reduce drug dose(s) and monitor with clinical follow-up within 1–2 weeks.
